# Factors associated with the inflammatory process in pain in ankylosing spondylitis

**DOI:** 10.11604/pamj.2022.41.331.31312

**Published:** 2022-04-25

**Authors:** Kaouther Maatallah, Yasmine Makhlouf, Hanene Ferjani, Ines Cherif, Dorra Ben Nessib, Wafa Triki, Dhia Kaffel, Wafa Hamdi

**Affiliations:** 1 Kassab Institute of Orthopedics, Tunis, Tunisia

**Keywords:** Ankylosing spondylitis, sleep disorders, anxiety, depression

## Abstract

**Introduction:**

sleep disorders, closely related to any chronic pain process, are frequent among patients with rheumatic diseases, mainly ankylosing spondylitis (AS). Our study aimed to determine the association between sleep disturbances and the inflammatory process in pain in AS patients compared with lower back pain (LBP) patients. We have additionally examined factors associated with sleep disorders among AS patients.

**Methods:**

we conducted a cross-sectional study among AS patients. Sociodemographic data, patient reported outcomes and disease characteristics were recorded. Sleep was assessed using the medical outcomes study sleep scale measure (MOS-SS). For psychological assessment, Beck anxiety (BAI) and depression index (BDI) was used. A multivariate logistic regression was performed to identify factors associated with sleep disorders.

**Results:**

the study included 50 patients with AS and 40 patients with low back pain. The most common affected domains among AS patients were inadequacy, sleep disturbance, and daily somnolence. The MOS-SS index was significantly higher in the AS group than in the control group (p<0.001). Sleep disorder was associated with age, female gender, analphabetism, patient-reported outcomes (all p<0.05), but was not associated with profession, comorbidities and smoking habits. In multivariate analysis, factors associated with sleep disruption were the duration of morning stiffness (MS), disease activity, bath ankylosing spondylitis metrology index (BASMI), ASQol, as well as anxiety and depression (odds ratio: 5.4(CI 95% 1.6-18.3), 9.9 (CI95%1.1-86); 6 (CI95%1.1-32); 13 (CI 95% 1.4-143.8); 15.7 (CI 95% 2.6-94.3); 14 (CI 95% 2-105.7) respectively, p<0.05 for each).

**Conclusion:**

our study highlighted the importance of sleep disorders among patients with AS with a predilection for inadequacy, sleep disturbance, and daily somnolence. Factors associated with sleep disruption were high disease activity, a longer duration of MS, an altered function and quality of life as well as anxiety and depression.

## Introduction

Ankylosing spondylitis (AS) is a systemic chronic inflammatory disease with unknown etiology. Because of the axial involvement, AS can lead to functional impairment and irreversible structural changes. Sleep disorders, closely related to any chronic pain process, are frequent among AS patients with a prevalence ranging between 50% and 64.5% [1]. These patients suffer from fragmented sleep because of their inflammatory low back involvement that occurs as axial pain, especially in the second half of the night. Morning stiffness, difficulty waking up, limited chest expansion, or obstructive sleep apnea syndrome (OSA) may lead to sleep disorders. Indeed, the incidence of OSA in AS patients is three times more frequent than in the general population [2]. A restriction of the oropharyngeal airway because of temporomandibular joint involvement, or ossification of the anterior longitudinal ligament may cause the latter [3]. Sleep is essential to keep the normal status of emotional, mental, and physical health. Indeed, sleep disorder was found to be associated with worsened function, fatigue, increased disease activity, and a lowered pain threshold [4,5]. Besides, depression and anxiety also play a significant role in sleep disruption among AS patients [6]. Non-specific chronic low back pain (LBP) is also known to cause sleep problems (50% of cases) [7]. Recent research showed that patients with LBP who reported sleep disturbance were twice as likely to be hospitalized compared to those who did not [8]. Unlike the inflammatory profile of the AS pain syndrome, LBP results in mechanical pain with increased nociceptive sensitivity. Despite the relevance of this topic, the impact of pain on sleep, according to its mechanism, has rarely been assessed in the literature.

This study aimed to determine the association between sleep disturbances and the inflammatory process in pain in AS patients compared with LBP patients to delineate the part of the inflammatory pain on sleep disorders. We have additionally examined factors associated with sleep disorders among AS patients.

## Methods

**Study design and setting:** we conducted a cross-sectional study carried out in our rheumatology department (Kassab Institute, Tunis, Tunisia) for twelve months.

**Study population:** we recruited participants with established AS according to the modified New York criteria between the period of 2019 and 2020. We did not include patients under the age of 18, with hearing or comprehension problems, with a history of malignancy or fibromyalgia, and patients taking medication that may affect the sleeping process. A control group was considered to ensure the validity of our study. It includes 40 patients with common LBP for more than 3 months.

**Data collected:** at inclusion, we recorded clinical data including age, marital status, level of education and profession. We also recorded smoking habits, family history of rheumatic diseases, and the body mass index (BMI) calculated as kg/height m^2^.

### Definitions

**Patient-reported outcomes:** we included the age of onset of the disease, the duration of the morning stiffness (MS), the number of night awakenings (NA), and pain via a ten-point incremental visual analogue scale (VAS). The bath ankylosing spondylitis-global score (BASG-s) was also measured; patients were asked to score the global impact of the disease on their well-being over the last week/6 months before the visit via a visual analogue scale (VAS) scale. BASG-s corresponds to the mean of the two scores. A lower score reflects a good overall condition of the patient.

**Disease activity:** disease activity was evaluated with the bath ankylosing spondylitis disease activity index (BASDAI) and the ankylosing spondylitis disease activity score: ASDAS ESR or ASDAS CRP [9,10].

**Enthesitis:** the Maastricht ankylosing spondylitis enthesitis score (MASES) was used to assess clinical enthesitis [11].

**Function:** the function was evaluated with the bath ankylosing spondylitis functional index (BASFI) and the bath ankylosing spondylitis metrology index (BASMI) [12,13].

**Quality of life:** the ankylosing spondylitis quality of life (ASQoL), we used the validated Tunisian version [14].

**Structural damage:** the bath ankylosing radiologic index (BASRI): this index allows the evaluation of structural damage during AS [15]. The modified stoke ankylosing spondylitis spine score (mSASSS): this score is used for quantitative assessment of axial damage [16].

**Psychological assessment:** for both groups, we evaluated anxiety and depression according to the Beck anxiety index (BAI) and the Beck depression index (BDI) respectively [17,18]. These self-questionnaires comprise 21 items each rated from 0 to 3. Anxiety is considered minimal between 0 and 17, moderate between 18 and 24, and severe beyond 25. Depression is considered minimal if the score varies between 0 and 3, mild between 4 and 7, moderate between 8 and 15, and severe beyond 16.

**Fatigue assessment:** the Chalder fatigue questionnaire (CFQ) provides a two-dimensional assessment of fatigue: somatic and mental. This score varies from 0 to 14. A score greater than 4 indicates fatigue [19].

**Sleep assessment:** the MOS-SS: medical outcomes study sleep scale measure (MOS-SS). This instrument evaluates sleep outcomes over the last 4 weeks. It comprises 12 items in six different subscales: “sleep disturbance”, “daytime somnolence”, “sleep adequacy”, “snoring”, “awaken short of breath or with headache” and “quantity of sleep/optimal sleep”. Ten of the 12 MOS-SS items are scored on a six-point categorical scale ranging from “all the time” to “none of the time” [20]. The scale also includes two indexes: the sleep problems index I that allows for the summary of sleep problems using an abbreviated six-item index (items 4, 5, 7, 8, 9, 12); and the sleep problems index II that uses 9 of the 12 items in the scale to compute an overall sleep problem summary. Higher scores for the domains show worse sleep problems [20].

**Statistical analysis:** data was captured using Excel software and analyzed using Statistical Package for Social Science (SPSS) software. The comparison of two independent series averages was made using the ANOVA test. The independent series percentage comparisons were made by Pearson's Chi-2 test, and in case of significance by the Chi-square test. In case of invalidity of the latter, the exact bilateral Fisher test was used. We then analyzed the relationship between AS characteristics and sleep disorder compared to patients with LBP. Median values of the control group were used as a cut-off to determine adjusted odds ratios (OR). To identify factors linked independently to the event, we conducted a multivariate logistic regression analysis step-by-step, odds ratios (OR) were calculated with a confidence interval (CI) fixed at 95%. The level of significance was fixed at p<0.05.

**Ethical considerations:** written informed consent was obtained from each patient. This study was approved by the Human Research Ethics in Kassab Institute of Orthopedics (18/20).

## Results

**General characteristics of the study population:** fifty patients with AS and 40 patients with LBP were enrolled in the study. There was a male predominance (78%, n=39). The mean age of AS and LBP pain group was 42 (SD 12.2) years (18-65) and 46.4 (SD 13.8) years (19-73) respectively. Among AS patients, a low level of education was found in 44% of cases (n=22); with 8% of them illiterate (n=4). Most of the patients were married (56%, n=28), unemployed (62%, n=31). The mean BMI was 24.8 (SD 4.1) Kg/m^2^ (17.3-39.8). Regarding treatment modalities, most of the patients (92%, n=46) were on non-steroidal anti-inflammatory drugs (NSAIDs). Sulfasalazine was prescribed in 13 patients and anti-TNF in 8 patients: etanercept (n=5), adalimumab (n=2), infliximab (n=1). The characteristics of AS patients are represented in Table 1.

**Table 1:** clinical, biological, and radiographic characteristics of patients with ankylosing spondylitis

Characteristics of AS patients	Value (mean (SD), or percentage and extremes)
Disease duration (years)	9.8 (SD 8.2) (0.5-30)
The mean age	42 (SD 12.2) (18-65)
Age of onset (years)	31.7 (SD 11.5) (16-58)
Comorbidities n (%)	11 (22)
Extra-articular manifestations n (%)	7 (14)
Coxitis n (%)	18 (36)
Night awakenings (number)	1.5 (SD 1.4) (0-5)
Morning stiffness (minutes)	36 (SD 35.9) (0-120)
VAS pain mean (mm)	49.8 (SD 25.1) (0.5-90)
BASG-s mean (0-100)	55.7 (SD 23.6) (5-90)
BASMI mean (0-10)	5 (SD 2.4) (1-10)
MASES mean (0-13)	1.6 (SD 2.3) (1-11)
BASFI mean (0-10)	4.8 (SD 2.9) (0-9.8)
ASQoL mean (0-18)	4.7 (SD 5.3) (0-17)
BASDAI mean (0-10)	4.7 (SD 2.4) (0-10)
**ASDAS**	
ESR mean	3 (SD 1.14) (1-5.1)
CRP mean	2.9 (SD 1) (1-5)
ESR mean (mm/h)	34.9 (SD 21.9) (4-84)
CRP mean(mg/l)	18.9 (SD 17) (3-73)
mSASSS mean (0-76)	10.5 (SD 13.3) (1-60)
BASRI mean (0-16)	7.5 (SD 3.2) (2-16)

AS: ankylosing spondylitis; SD: standard deviation; n: number of patients; VAS: visual analogue scale; BASMI: bath ankylosing spondylitis metrology index; BAS-G: bath ankylosing spondylitis -global score; MASES: Maastricht ankylosing spondylitis enthesitis score; BASDAI: bath ankylosing spondylitis disease activity index; ASDAS: ankylosing spondylitis disease activity score; ESR: erythrocyte sedimentation rate; CRP: C-reactive protein; BASFI: bath ankylosing spondylitis functional index; ASQo: ankylosing spondylitis quality of life; mSASSS: modified stoke ankylosing spondylitis spine score, BASRI: bath ankylosing radiologic index

**Comparison of sleep disorders between AS and LBP patients:** all the MOS-SS domains were altered with a predilection for inadequacy, sleep disturbance, and daily somnolence. Half of AS patients slept less than 6 hours. The mean scores for each subset were significantly higher in AS patients compared with LBP except for quantity of sleep and snoring. Among AS patients, the BDI/BAI scores were as follows: minimal (12% (n=6)/72% (n=36)), mild (18% (n=9)/none), moderate (22% (n=11)/6% (n=3), and severe (48%/22%) respectively. Fatigue was found in most of the cases (84%, n=42). The comparison of the different outcome measures between the AS and LBP patients is represented in Table 2.

**Table 2:** evaluation of fatigue, psychological status, and sleep disorders in the study and control group

		Study group (AS) n=50	Control group (LBP) N=40	p
Anxiety depression	BDI mean	16.2 (SD 11.9) (0-43)	5.6 (SD 5.6) (0-23)	<0.001
	BAI mean	14.8 (SD 10.5) (0-36)	6.3 (SD 5.6) (0-22)	<0.001
Fatigue	Global fatigue	7.9 (SD 3.5) (0-14)	4.9 (SD 2.9) (0-13)	<0.001
	Organic fatigue	5.9 (SD 2.3) (0-8)	4 (SD 1.9) (0-7)	<0.001
	Mental fatigue	1.8 (SD 2) (0-6)	0.8 (SD 1.4) (0-6)	0.017
MOS-SS	Sleep adequacy	53.2 (SD 23.7) (10-90)	21.3 (SD 21) (0-80)	<0.001
	Sleep disturbance	49.7 (SD 25.6) (0-100)	26 (SD 22.9) (0-85)	<0.001
	Daytime somnolence	52.9 (SD 20.2) (0-93.3)	19.1 (SD 17.2) (0-60)	<0.001
	Snoring	35.8 (SD 32.8) (0-100)	29.3 (SD 22.5) (0-100)	0.293
	Headache	32.2 (SD 30.6) (0-100)	15 (SD 33.6) (0-80)	0.003
	Quantity of sleep	5.5 (SD 1.5) (2-10)	5.1 (SD 1.3) (2-8)	0.215
SP	SPI	51.6 (SD 22.7) (10-90)	19.2 (SD 18.7) (0-76.6)	<0.001
	SPII	50.9 (SD 21.2) (9.4-96.1)	21.2 (SD 19) (0-80)	<0.001

BDI: Beck depression index; BAI: Beck anxiety index; MOS-SS: medical outcomes study sleep scale measure; SP: sleep problems

**Determinants of sleep disorders in the AS group:** in univariate analysis, sleep disorders were correlated to age, female gender, analphabetism, and the lack of social security (Table 3). We found no association between sleep disruption and the other sociodemographic data (living environment, socioeconomic level, profession, comorbidities, or smoking). Regarding disease characteristics, disease duration was negatively correlated with sleep quantity, with no influence on other sleep domains. Patient-reported outcomes (NA, MS, VAS) were positively correlated with sleep disturbance, daytime somnolence, sleep adequacy, headache, SPI, and SPII and negatively correlated with sleep quantity. Similarly, there was a correlation between sleep disturbances and disease activity as well as function. In multivariate analysis, factors associated with sleep disruption were the duration of MS, disease activity, ASQol, BASMI as well as anxiety and depression (Table 4).

**Table 3:** study of the association between sleep disorders and sociodemographic data in the ankylosing spondylitis group in univariate analysis

	Age	Gender	Level of education	Marital status	BMI
Sleep disturbance	p=0.015	p=0.536	p=0.883	p=0.316	p=0.819
Snoring	p=0.011	p=0.977	p=0.947	p=0.030	p=0.036
Headache	p=0.051	p=0.037	p=0.062	p=0.201	p=0.496
Sleep adequacy	p=0.124	p=0.287	p=0.019	p=0.475	p=0.616
Daytime somnolence	p=0.084	p=0.685	p=0.305	p=0.239	p=0.684
Quantity of sleep	p=0.092	p=0.634	p=0.032	p=0.388	p=0.979
SPI	p=0.041	p=0.240	p=0.329	p=0.102	p=0.642
SPII	p=0.046	p=0.257	p=0.195	p=0.172	p=0.844

BMI: Body mass index, SP: sleep problems

**Table 4:** univariate and multivariate analysis for predicting factors of sleep disturbances in AS patients

	Univariate analysis		Multivariate analysis	
Variables	r	p	HR (95% CI)	P value
Morning stiffness	0.346	0.014	5.4 (1.6-18.3)	<0.05
BASDAI	0.444	0.006	9.9 (1.1-86)	<0.05
BASMI	0.4	0.05	6 (1.1-32)	<0.05
ASQol	0.345	0.04	13 (1.4-143.8)	<0.05
BAI	0.574	0.000	15.7 (2.6-94.3)	<0.05
BDI	0.656	0.000	14(2-105.7)	<0.05
ESR	-0.320	0.019	5.4 (1-28.7)	p>0.05
CRP	0.405	0.034	12.5(1.9-109.6	p>0.05
VAS	0.460	0.001	8.5 (2.3-31)	>0.05
BASFI	0.604	0.000	3.7 (1.1-12.1)	>0.05
BASG	0.588	0.000	16.7 (1.4-145.7)	>0.05
BASRI	0.08	0.552	-	-

AS: ankylosing spondylitis; VAS: visual analogue scale; BASFI: bath ankylosing spondylitis functional index; ESR: erythrocyte sedimentation rate; CRP: C-reactive protein; BASDAI: bath ankylosing spondylitis disease activity index; BASMI: bath ankylosing spondylitis metrology index; ASQol: ankylosing spondylitis quality of life; BAI: Beck anxiety index; BDI: Beck depression index; CI: confidence interval

## Discussion

The main objective of our study was to determine the association between sleep disturbances and the inflammatory process in pain in AS patients compared with LBP patients. Our study showed that sleep disorders are common among AS patients, affecting all the domains with a predilection for inadequacy, sleep disturbance, and daily somnolence. The prevalence of sleep disorders has been reported to occur in 50 to 64.5% of AS patients; varying according to the sleep test [1]. Regarding mechanical LBP, the main factor affecting sleep is pain. A bidirectional relationship between pain and sleep quality was reported [21]. Indeed, Alsaadi *et al*. showed that for every decrease in sleep quality, pain upon waking is 1-point higher on the VAS. Also, for every increase in average daytime pain intensity, there is a 0.20-point decrease in sleep quality [21]. Unlike AS, few studies focused on the extent to which sleep quality is influenced by other pain-related outcomes such as psychological status and function. In a study, disturbance of the quality of sleep-in individuals with LBP was correlated with physical disability [22]. In another study, poor sleep was related to pain intensity, physical function, and higher levels of negative affect [23].

A recent study showed that decreased central processing of pain varied between individuals with LBP and was related to factors such as sleep and alcohol consumption [24]. More importantly, our findings support the role of the inflammatory processes in understanding the bidirectional association between pain and sleep. Previous research has showed that inflammatory mediators can modulate nociception and are responsible for central sensitization [25]. Indeed, higher levels of cytokines especially interleukin-6 are associated with pain severity in patients with rheumatoid arthritis and fibromyalgia [26]. In our study, age, female gender, and analphabetism were the only sociodemographic factors correlated with sleep disorders. Moreover, obese patients had higher snoring scores. However, literature studies showed that there is no influence of the BMI on the occurrence of OSA during AS [27,28], but its prevalence increased in patients with an advanced cervical vertebral disease [27,29]. The use of steroids with an increase in neck circumference and body weight has also been proposed as other mechanisms of sleep disorders in AS [27]. Data on age was consistent with our results and showed that age was correlated with sleep disorders regardless of the used questionnaire [29,30]. However, it is important to consider that sleep naturally changes with older age as slow-wave sleep decreases.

Data on gender effect on sleep was conflicting. While some studies do not support a marked gender difference in sleep disruption for AS population [31-33], other studies were consistent with our results [34,35]. Indeed, Hultgren *et al*. showed that among 70 AS patients, 81% of female patients reported not getting enough sleep, compared to 50% of male patients [35]. In line with our results, a recent review of the literature reported that sleep quality in patients with AS is most associated with age and gender [36]. Similar to our findings, a lower level of education was an important predictor of impairment of the different components of quality of life, including sleep according to Ward *et al*. [37]. On the contrary, Li *et al*. showed that poor sleep quality had an inverse relationship with the number of years in education [38]. Regarding patient-reported outcomes, no correlation was found between the quality of sleep and disease duration [39,40]. Abdulaziez *et al*. found a significant correlation between sleep disturbance and the disease duration [36].

During the early stages of AS, nociceptive pain and inflammation may affect sleep. However, in advanced stages, functional discomfort and stiffness are the main complaints rather than pain. At this stage, the impact of sleep is less important as patients adapt to their new physical state. In a recent meta-analysis, no significant associations were found between age, gender, disease duration BASDAI, BASMI, and each domain of the Pittsburgh sleep quality index (PSQI) [41]. The increased use of sleep medication in recent studies may explain these findings and might be a publication bias [41]. BASMI was also found to be correlated with sleep disturbances [30,32,36,41]. Indeed, reduced spinal mobility leads to difficulties in changing positions during sleep, and thus inadequate sleep and daytime sleepiness. In contrast, Li *et al*. found no relationship between BASMI and sleep problems assessed by the PSQI. This may be explained by the low average of BASMI in the study population [38].

Similarly, a significant correlation has been demonstrated between ASQoL and most of the MOS-SS domains, which was consistent with several studies [36,38,42]. To our knowledge, this is the first study assessing the effect of enthesitis involvement and ASDAS on sleep disorders. Regarding psychological status, anxiety and depression were identified as factors associated with sleep disorders. A review of the literature showed that anxiety and depression were associated with poor quality of sleep [36]. Li *et al*. showed a higher frequency of depressive disorders in the group of poor sleepers [38]. Similarly, reduced sleep duration activates the hypothalamic-pituitary-adrenal axis and induces a stress response, which maintains a depressive state [43]. The relationship between sleep and anxiety is less studied compared to the relationship between depression and sleep. Curiously, Augner *et al*. studied separately anxiety as a personality trait and illness and found that anxiety as a personality trait was correlated with sleep [44].

The strength of our work is that it is the first study assessing the impact of pain on sleep, according to its mechanism. Instead of healthy volunteers, the control group included patients with non-specific LBP. This enabled us to distinguish between the impact of inflammatory and mechanical pain. Indeed, the sleep disorder in AS was not only determined by the intensity of the pain (like in LBP), but also by disease activity and the duration of morning stiffness which highlights the role of the inflammatory process. We used several validated self-questionnaires that have good reproducibility including the assessment of enthesitis with MASES. Nevertheless, this study had some limitations and the primary one being its cross-sectional design. A longitudinal study may be interesting as psychological status, sleep and disease activity can be monitored. Moreover, sleep disorders should be assessed during active and non-active periods.

## Conclusion

Our study highlighted that sleep disorders are common among AS patients and mainly attributed to the inflammatory process of pain. Factors associated with sleep disruption were high disease activity, a longer duration of MS, an altered function and quality of life as well as anxiety and depression. Screening for these may help identify a patient group that is at risk of developing chronic sleep problems and investigating this relationship may provide directions for the design of interventions to manage these conditions.

### What is known about this topic

- Sleep disorders, closely related to any chronic pain process, are frequent among patients with rheumatic diseases mainly ankylosing spondylitis;
- The relationship between sleep and pain whether it is mechanical or inflammatory is complex and multifactorial.

### What this study adds

- The inflammatory process of pain is the main factor incriminated in sleep disorders among AS patients;
- Factors associated with sleep disruption were high disease activity, altered function and quality of life as well as anxiety and depression.

## References

[1] Aydin E, Bayraktar K, Turan Y, Omurlu I, Tastaban E, Sendur OF (2015). Sleep quality in patients with ankylosing spondylitis. Rev Bras Reumatol.

[2] Tsao CH, Huang JY, Huang HH, Hung YM, Wei JC, Hung YT (2019). Ankylosing spondylitis is associated with risk of new-onset obstructive sleep apnea: a nationwide population-based cohort study. Front Med (Lausanne).

[3] Wang Y, Lin S, Li C, Shi Y, Guan W (2020). Sleep apnea-hypopnea syndrome caused by ankylosing spondylitis: a case report. Medicine (Baltimore).

[4] Jiang Y, Yang M, Wu H, Song H, Zhan F, Liu S (2015). The relationship between disease activity measured by the BASDAI and psychological status, stressful life events, and sleep quality in ankylosing spondylitis. Clin Rheumatol.

[5] Jiang Y, Yang M, Lv Q, Qi J, Lin Z, Liao Z (2018). Prevalence of psychological disorders, sleep disturbance, and stressful life events and their relationships with disease parameters in Chinese patients with ankylosing spondylitis. Clin Rheumatol.

[6] Zou Q, Jiang Y, Mu F, Shi Y, Fang Y (2016). Correlation of axial spondyloarthritis with anxiety and depression. Med Sci Monit.

[7] Silva HJA, Saragiotto BT, Silva RS, Lins CAA, de Souza MC (2019). Dry cupping in the treatment of individuals with non-specific chronic low back pain: a protocol for a placebo-controlled, randomized, double-blind study. BMJ Open.

[8] Alsaadi SM, McAuley JH, Hush JM, Maher CG (2011). Prevalence of sleep disturbance in patients with low back pain. Eur Spine J.

[9] Garrett S, Jenkinson T, Kennedy LG, Whitelock H, Gaisford P, Calin A (1994). A new approach to defining disease status in ankylosing spondylitis: the bath ankylosing spondylitis disease activity index. J Rheumatol.

[10] Lukas C, Landewe R, Sieper J, Dougados M, Davis J, Braun J (2009). Development of an ASAS-endorsed disease activity score (ASDAS) in patients with ankylosing spondylitis. Ann Rheum Dis.

[11] Heuft-Dorenbosch L, Spoorenberg A, van Tubergen A, Landewé R, van ver Tempel H, Mielants H (2003). Assessment of enthesitis in ankylosing spondylitis. Ann Rheum Dis.

[12] Calin A, Garrett S, Whitelock H, Kennedy LG, O'Hea J, Mallorie P (1994). A new approach to defining functional ability in ankylosing spondylitis: the development of the bath ankylosing spondylitis functional index. J Rheumatol.

[13] van der Heijde D, Landewé R, Feldtkeller E (2008). Proposal of a linear definition of the bath ankylosing spondylitis metrology index (BASMI) and comparison with the 2-step and 10-step definitions. Ann Rheum Dis.

[14] Hamdi W, Haouel M, Ghannouchi MM, Mansour A, Kchir MM (2012). Validation of the ankylosing spondylitis quality of life questionnaire in Tunisian language. Tunis Med.

[15] MacKay K, Mack C, Brophy S, Calin A (1998). The bath ankylosing spondylitis radiology index (BASRI): a new, validated approach to disease assessment. Arthritis Rheum.

[16] Creemers MC, Franssen MJ, van´t Hof MA, Gribnau FW, van de Putte LB, van Riel PL (2005). Assessment of outcome in ankylosing spondylitis: an extended radiographic scoring system. Ann Rheum Dis.

[17] Beck AT, Epstein N, Brown G, Steer RA (1988). An inventory for measuring clinical anxiety: psychometric properties. J Consult Clin Psychol.

[18] Beck AT, Steer RA, Garbin MG (1988). Psychometric properties of the Beck depression inventory: twenty-five years of evaluation. Clinical Psychology Review.

[19] Chalder T, Berelowitz G, Pawlikowska T, Watts L, Wessely S, Wright D (1993). Development of a fatigue scale. J Psychosom Res.

[20] Hays RD, Martin SA, Sesti AM, Spritzer KL (2005). Psychometric properties of the medical outcomes study sleep measure. Sleep Med.

[21] Alsaadi SM, McAuley JH, Hush JM, Lo S, Bartlett DJ, Grunstein RR (2014). The bidirectional relationship between pain intensity and sleep disturbance/quality in patients with low back pain. Clin J Pain.

[22] McCracken LM, Iverson GL (2002). Disrupted sleep patterns and daily functioning in patients with chronic pain. Pain Res Manag.

[23] Gerhart JI, Burns JW, Post KM, Smith DA, Porter LS, Burgess HJ (2017). Relationships between sleep quality and pain-related factors for people with chronic low back pain: tests of reciprocal and time of day effects. Ann Behav Med.

[24] Klyne DM, Moseley GL, Sterling M, Barbe MF, Hodges PW (2018). Individual variation in pain sensitivity and conditioned pain modulation in acute low back pain: effect of stimulus type, sleep, and psychological and lifestyle factors. J Pain.

[25] Morris P, Ali K, Merritt M, Pelletier J, Macedo LG (2020). A systematic review of the role of inflammatory biomarkers in acute, subacute and chronic non-specific low back pain. BMC Musculoskelet Disord.

[26] Heffner KL, France CR, Trost Z, Ng HM, Pigeon WR (2011). Chronic low back pain, sleep disturbance, and interleukin-6. Clin J Pain.

[27] Erdal IN, Turgut T, Gülkesen A, Gündogdu B (2015). Obstructive sleep apnea syndrome and sleep efficiency in patients with ankylosing spondylitis. Arch Rheumatol.

[28] Erb N, Karokis D, Delamere JP, Cushley MJ, Kitas GD (2003). Obstructive sleep apnoea as a cause of fatigue in ankylosing spondylitis. Ann Rheum Dis.

[29] Solak O, Fidan F, Dundar U, Türel A, Ayçiçek A, Kavuncu V (2009). The prevalence of obstructive sleep apnoea syndrome in ankylosing spondylitis patients. Rheumatology (Oxford).

[30] Abdulaziez O, Asaad T (2012). Sleep problems in ankylosing spondylitis: polysomnographic pattern and disease-related variables. Egyptian Rheumatologist.

[31] Hakkou J, Rostom S, Mengat M, Aissaoui N, Bahiri R, Hajjaj Hassouni N (2013). Sleep disturbance in Moroccan patients with ankylosing spondylitis: prevalence and relationships with disease-specific variables, psychological status and quality of life. Rheumatol Int.

[32] Da Costa D, Zummer M, Fitzcharles MA (2009). Determinants of sleep problems in patients with spondyloarthropathy. Musculoskeletal Care.

[33] Heiberg T, Lie E, van der Heijde D, Kvien TK (2011). Sleep problems are of higher priority for improvement for patients with ankylosing spondylitis than for patients with other inflammatory arthropathies. Ann Rheum Dis.

[34] López-Medina C, Schiotis RE, Font-Ugalde P, Castro-Villegas MC, Calvo-Gutiérrez J, Ortega-Castro R (2016). Assessment of fatigue in spondyloarthritis and its association with disease activity. J Rheumatol.

[35] Hultgren S, Broman J, Gudbjörnsson B, Hetta J, Lindqvist U (2000). Sleep disturbances in outpatients with ankylosing spondylitis a questionnaire study with gender implications. Scand J Rheumatol.

[36] Leverment S, Clarke E, Wadeley A, Sengupta R (2017). Prevalence and factors associated with disturbed sleep in patients with ankylosing spondylitis and non-radiographic axial spondyloarthritis: a systematic review. Rheumatol Int.

[37] Ward MM (1999). Health-related quality of life in ankylosing spondylitis: a survey of 175 patients. Arthritis Care Res.

[38] Li Y, Zhang S, Zhu J, Du X, Huang F (2012). Sleep disturbances are associated with increased pain, disease activity, depression, and anxiety in ankylosing spondylitis: a case-control study. Arthritis Res Ther.

[39] Karadag O, Nakas D, Kalyoncu U, Akdogan A, Kiraz S, Ertenli I (2012). Effect of anti-TNF treatment on sleep problems in ankylosing spondylitis. Rheumatol Int.

[40] Urkmez B, Keskin Y (2020). Relationship between sleep quality and physical activity level in patients with ankylosing spondylitis. Mod Rheumatol.

[41] Li Z, Fu T, Wang Y, Dong C, Shao X, Li L (2019). Sleep disturbances in ankylosing spondylitis: a systematic review and meta-analysis. Psychol Health Med.

[42] Batmaz I, Sariyildiz M, Dilek B, Bez Y, Karakoc M, Cevik R (2013). Sleep quality and associated factors in ankylosing spondylitis: relationship with disease parameters, psychological status and quality of life. Rheumatol Int.

[43] Baysal O, Durmus B, Ersoy Y, Altay Z, Senel K, Nas K (2011). Relationship between psychological status and disease activity and quality of life in ankylosing spondylitis. Rheumatol Int.

[44] Augner C (2011). Associations of subjective sleep quality with depression score, anxiety, physical symptoms and sleep onset latency in students. Cent Eur J Public Health.

